# An ounce of prevention is better: Monitoring wildlife health as a tool for pandemic prevention

**DOI:** 10.1038/s44319-024-00156-z

**Published:** 2024-06-07

**Authors:** Jan Frederik Gogarten, Ariane Düx, Tobias Gräßle, Christelle Patricia Lumbu, Stephanie Markert, Livia Victoria Patrono, Kamilla Anna Pléh, Frederic Niatou Singa, Coch Tanguy Floyde Tanga, Thais Berenger Tombolomako, Emmanuel Couacy-Hymann, Leonce Kouadio, Steve Ahuka-Mundeke, Patrice Makouloutou-Nzassi, Sébastien Calvignac-Spencer, Fabian Hubertus Leendertz

**Affiliations:** 1 Helmholtz Institute for One Health, Helmholtz-Centre for Infection Research (HZI), Greifswald, Germany; 2 Department of Applied Zoology and Nature Conservation, University of Greifswald, Greifswald, Germany; 3 Taï Chimpanzee Project, Centre Suisse de Recherches Scientifiques, Abidjan, Côte d’Ivoire; 4 Institut National de Recherche Biomedical, Kinshasa, Democratic Republic of the Congo; 5 Kokolopori Bonobo Research Project, Tshuapa, Democratic Republic of the Congo; 6 WWF Central African Republic Programme Office, Dzanga Sangha Protected Areas, Bangui, Central African Republic; 7 Institut de Recherche en Ecologie Tropicale, Libreville, Gabon; 8 Ozouga Chimpanzee Project, Loango National Park, Gabon; 9 Loango Gorilla Project, Loango National Park, Gabon; 10 Centre National de Recherche Agronomique (CNRA), Abidjan, Côte d’Ivoire; 11 Université Peleforo Gon Coulibaly, Korhogo, Côte d’Ivoire; 12 Centre Suisse de Recherches Scientifiques, Abidjan, Côte d’Ivoire; 13 Service de Microbiologie, Departement de Biologie Médicale, Cliniques Universitaires de Kinshasa (CUK), Université de Kinshasa (UNIKIN), Kinshasa, Democratic Republic of the Congo; 14 Institut National de Recherche Biomédicale, Kinshasa, Democratic Republic of the Congo; 15 Département de Biologie et Écologie Animale, Institut de Recherche en Écologie Tropicale (IRET/CENAREST), Libreville, Gabon; 16 Unité de Recherche en Écologie de la Santé, CIRMF, Franceville, Gabon; 17 Faculty of Mathematics and Natural Sciences, University of Greifswald, Greifswald, Germany

**Keywords:** Evolution & Ecology, Microbiology, Virology & Host Pathogen Interaction

## Abstract

Long-term observations of wildlife are key to understanding the ecological foundations of disease emergence. They provide unique opportunities to detect pathogens with zoonotic potential that could threaten human health but also pose a threat for the animals.

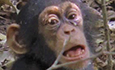

Emerging infectious diseases (EIDs) have exacted an enormous toll on humanity for millennia. Owing to human activities, however, our planet is changing at an unprecedented rate, which heightens the risk of new diseases emerging; indeed, EIDs have been on the rise since, at least, the 1940s (Jones et al, 2008). While various factors contribute to this increase, anthropogenic disturbance and changes in land use have led to broader interfaces with wildlife, the main source of emerging pathogens (Jones et al, 2008; Allen et al, 2017). Human-dominated ecosystems also have a greater diversity and abundance of wildlife species known to host zoonotic agents (Gibb et al, 2020). Despite their importance, these interfaces are generally poorly understood, and the specific factors that contributed to the emergence of pathogens remain unclear. For example, the origins of the Ebola virus epidemic in West Africa between 2014 and 2016 that claimed the lives of at least 11,325 people is still unclear. Although there are hypotheses about the source, the virus was not detected in wildlife and the exact ecological and social context in which the outbreak began remains contentious.

Owing to human activities, however, our planet is changing at an unprecedented rate, which heightens the risk of new diseases emerging; indeed, EIDs have been on the rise since, at least, the 1940s.

Considerable emphasis has been placed on containing EIDs: reducing the impact of spillover events by reducing EID spread in human populations. Such efforts are necessary, particularly near biodiversity hotspots that are also disease-emergence hotspots. Yet, it is also clear that decreasing the likelihood of spillovers in the first place will also be necessary to mitigate the threat (OHLEP et al, 2023). To achieve effective prevention, a comprehensive understanding of the ecological foundations of disease emergence is crucial. We argue that long-term observations of wildlife are essential for accumulating such knowledge. As an example of such efforts, we describe how African great-ape health-monitoring programs have contributed to pandemic prevention.

## A plea for longitudinal wildlife surveillance

Given the immense diversity of animals worldwide, it is impossible to characterize the zoonotic potential of each of the bacteria, viruses, and fungi they host.

Given the immense diversity of animals worldwide, it is impossible to characterize the zoonotic potential of each of the bacteria, viruses and fungi they host (Petrone et al, 2023). Current animal surveillance efforts tend to be short-term in nature and focus on a small subset of species in a given ecosystem. While efforts to develop predictive models to assess the zoonotic potential of microorganisms based solely on their genetic sequences are making headway (Mollentze and Streicker, 2023), sequencing the microorganism communities of representatives from all populations of animals on the planet is currently unfeasible. The challenging scope of these efforts is highlighted by the fact that, despite the enormous research efforts already conducted on our own species, we continue to discover new endemic, human-infecting pathogens.

Rather than trying to characterize all microorganisms on the planet, targeted surveillance is a more practical approach given the limited resources. Microorganisms capable of infecting multiple mammalian host species in an ecosystem have a higher potential to spill over into human populations (Petrone et al, 2023). Identifying such microorganisms requires pathogen surveillance efforts that encompass a broad range of animal species in their natural habitats. However, given the elusive nature of most wildlife species, such monitoring is often restricted to postmortem sampling or capturing animals. Here, we argue that longitudinal observation of species that are being extensively studied for their behavior, can allow for the detection of animals displaying visible signs of disease, which facilitates more targeted sampling. At the same time, these efforts provide information about the broader ecology of these animals, including their interactions with other host species. We argue that such longitudinal observation efforts increase our ability to identify microorganisms that are particularly relevant for human health and therefore, prevention.

## Coupling disease monitoring with longitudinal studies in wild great apes

Arguably one of the best examples for the usefulness of this approach comes from studies on wild nonhuman primates in their natural ecosystems, particularly those focusing on human’s closest phylogenetic relatives, the great apes (Calvignac-Spencer et al, 2021). Historically, nonhuman primates, in particular great apes, were considered the optimal proxies for studying the pathogenesis of human diseases, because we are immunologically and physiologically so similar. However, due to evident ethical concerns, such studies have become highly restricted or are entirely prohibited. Nevertheless, great apes living in the wild offer unique opportunities for longitudinal surveillance, which are particularly useful for detecting potentially zoonotic pathogens. A vast number of wild-living chimpanzees, gorillas, bonobos and orangutans have been habituated to human presence as part of ecotourism and research projects. This means they are—at least largely—undisturbed by human presence and can be observed in their natural habitats, providing an unparalleled opportunity for frequent monitoring of disease manifestations and mortality and concomitant sample collection. Combining infectious disease monitoring with primatology has proven a powerful strategy for understanding relevant pathogen diversity and epidemiology, as well as great-ape conservation, which we will illustrate in detail below.

Combining infectious disease monitoring with primatology has proven a powerful strategy for understanding relevant pathogen diversity and epidemiology, as well as great-ape conservation…

While early efforts to study great apes involved varied attempts to “civilize” them in captivity, the last half century has seen a huge proliferation of efforts to study them in the wild and to understand their natural behavior. Prominent examples of such pioneering efforts are the chimpanzees at Gombe, habituated by Jane Goodall, the mountain gorillas habituated by Dian Fossey, and Birute Galdikas’ work with Bornean orangutans, which have been scaled up with efforts such as the *Pan African Programme: The Cultured Chimpanzee*. These projects have continued over many decades, involving huge time investments by local populations and the long-term commitment of scientists and staff. They often have to overcome the significant hurdle that most nonhuman primates are initially very wary of humans, particularly in habitats where they are hunted. The habituation process can take many months for monkey species and even years for great apes, particularly after primatologists realized that feeding primates to get them used to humans, greatly alters their biology and behavior.

… most sampling of great apes must rely on noninvasive methods, such as observations from a distance, or the collection of feces, urine or discarded food remains with saliva on them.

Depending on the species, this familiarity is also not permanent, as many great apes are quick to remember a negative encounter—for instance, an injection from a blowpipe—which can set back habituation efforts and greatly reduce their comfort with people. As a result, most sampling of great apes must rely on noninvasive methods, such as observations from a distance, or the collection of feces, urine or discarded food remains with saliva on them. It is only in rare cases, when an animal dies and the carcass is found before scavengers and the rapid decomposition typical of the tropics make it disappear, that tissue samples can be collected. It is important to note that such collection of tissue samples is anything but straightforward, meaning that noninvasive samples represent the bulk of available material for laboratory analyses.

Habituation efforts provided exciting insights into wild great-ape life and behavior, but reports on diseases in these populations followed suit. For example, there were reports of diverse skin diseases and a large suspected polio outbreak in the chimpanzees in Gombe as early as in the 1960s. However, back then, samples were typically not collected for diagnostics, and the technological advances allowing noninvasive sampling for studying pathogens were decades away; thus, it took quite some time for systematic disease investigations and diagnostics to become integrated into primatological research.

## Carcass monitoring of great apes in their natural habitats

A pioneer in these efforts was the Taï Chimpanzee Project (TCP) in Côte d’Ivoire, which was initiated by Christophe Boesch and his team in 1979 (Boesch et al, 2019). There, roughly 25% of a 43-member chimpanzee community died in a dramatic disease outbreak in 1994 (Formenty et al, 1999). Unaware of the risks, researchers who were unequipped and untrained in infectiology and biosafety protocols tried to collect samples from a chimpanzee carcass and one of them became infected. Research into this human case and the chimpanzee samples that were collected led to the discovery of a new strain and species of Ebola virus (Taï Forest ebolavirus; TAFV). Fortunately, the researcher survived and represents the only known human case (Formenty et al, 1999). In an attempt to find the reservoir of the TAFV, a targeted investigation occurred in Taï National Park (hereafter simply: Taï) between 1996 and 2000. TAFV was never detected again, but it became clear that infectious diseases caused by other pathogens were responsible for a significant fraction of chimpanzee mortality.

**Figure 1:**
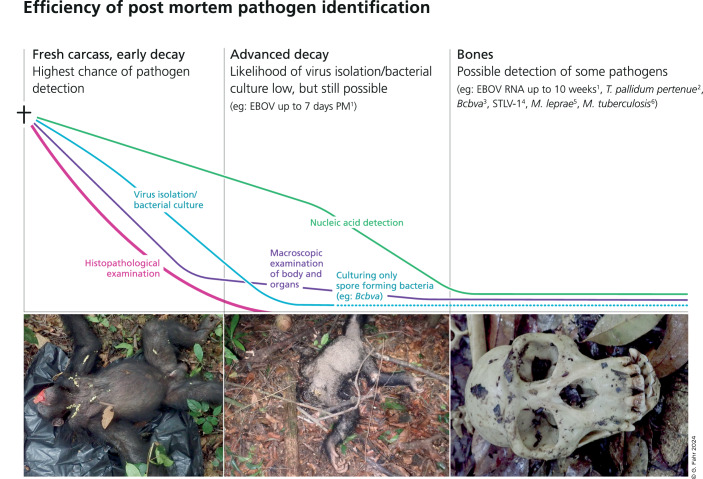
Efficiency of postmortem pathogen identification. When monitoring wildlife based on animal carcasses, the likelihood of pathogen detection is strongly influenced by decomposition, that is, diagnostic options become increasingly limited and less sensitive as the carcass decomposes. For example, while histopathology requires fresh tissues to evaluate cellular structures, nucleic acids of pathogens might be detected throughout and even after carcass putrefaction and skeletonization. Pathogen culture predominantly necessitates relatively fresh specimens; however, spore-forming bacteria may even be efficiently cultured from bone samples. Initially, a carcass is often mostly intact (left) and without evident signs of decomposition or rigor mortis, but flies can rapidly start laying their eggs even before an animal dies. At this early stage, samples from all organs can still be easily collected. After some time (middle), the carcass becomes bloated, internal organs decompose, and scavengers often begin feeding on it. Maggots (fly larvae) begin to appear and accelerate decomposition, and there is a strong odor. At this stage, it can sometimes still be possible to sample the remaining organs, but pathogens can also be detected in swabs or maggots collected from the carcass. At later stages of decomposition, when only hair and bones remain (right), maggots can sometimes still be present even after the flesh has decomposed and can be collected for testing. Otherwise, bones and bone swabs can be collected. The speed of decomposition is influenced by many environmental factors such as temperature, humidity, scavengers, or arthropods. The likelihood of detecting a pathogen shown in these curves thus represents a rough approximation based on our experience and published data on great apes, including humans, as well as from other animals (see Further Reading).

In 2000, the TCP, together with a team of local and global partners, began to systematically track the health of chimpanzees and other wildlife in the park. This included observing and describing disease manifestations in living individuals, but also full necropsies under rigorous safety standards when a chimpanzee or other animal carcass was found (Fig. 1). Collecting such samples is no small feat. It requires an infrastructure and materials, as well as trained personnel to be permanently in place in remote forest camps. It is not known in advance when a carcass will be found, and necropsies need to be performed on the spot as transporting carcasses represents too high a risk for pathogen spread. In Taï, huge efforts were made to bring liquid nitrogen into forest camps to allow for flash freezing of samples that were subsequently transported on dry ice. At other sites, where cold chains are impossible to maintain, sample preservation techniques that do not require freezing are now applied, such as RNAlater or Nucleic Acid Preservation Buffer. In the two decades since, a trained veterinarian has been on-site nearly year-round, and more than 400 necropsies have been safely performed. Indeed, since heeding the clear warning posed by the 1994 TAFV outbreak, no further human infections have occurred, highlighting the effectiveness of well-trained staff following stringent biosafety protocols in greatly minimizing risk.

Systematic carcass monitoring led to the discovery of a variety of pathogens that are potentially relevant to humans. For example, a novel type of anthrax-causing bacillus (*Bacillus cereus* biovar *anthracis*, *Bcbva*) was quickly discovered to be causing massive mortality in chimpanzees and other mammals, representing a potentially zoonotic disease that is likely fatal to humans if untreated (Hoffmann et al, 2017; Box 1). Leprosy and Mpox, the latter causing mortality, were first detected after two decades of observation (Hockings et al, 2021; Patrono et al, 2020; Boxes 2 and 3). Importantly, a number of human respiratory pathogens were also linked to severe outbreaks in wild chimpanzees (Köndgen et al, 2008; Box 4).

Systematic carcass monitoring led to the discovery of a variety of pathogens that are potentially relevant to humans.

This led to the implementation of strict hygiene protocols to limit the risk of spillover of pathogens from humans to animals. These include health monitoring and vaccination of staff, a 5-day quarantine in a separate camp prior to staff moving to other camps and observing chimpanzees, wearing masks when with the chimpanzees, maintaining a distance from animals and removing human feces from the forest. These protocols make work with great apes significantly more challenging and time-consuming, but as evidence continued to mount at Taï and other field sites they became the basis for the IUCN best-practice guidelines (Gilardi et al, 2015).

At the same time, these links between human and great-ape health highlighted the importance of a One Health framework for mitigating disease risk for both humans and chimpanzees. Consequently, public health work with the local community has become a major component of these efforts. Indeed, the detection of novel pathogens or distinct novel presentations can guide human surveillance efforts.

Box 1: **Anthrax** On-site necropsies had just been implemented in Taï, when a series of sudden chimpanzee deaths occurred in 2001. The dissections of the previously healthy chimps revealed hemorrhagic organs, foremost lungs (Fig A), pointing to lethal infectious events with Ebola hemorrhagic fever as one of the main suspects. However, the histological visualization of encapsulated bacteria throughout the organs (Fig B) and positive results in a PCR screening for *Bacillus (B.) anthracis* led to the unambiguous diagnosis of anthrax, a severe infectious disease that can be lethal for humans (Leendertz et al, 2004a). This was unexpected, since natural anthrax fatalities in wild chimpanzees had never been reported before.

Even more conspicuous was the fact that the isolated bacterium exhibited microbiological traits unusual for *B. anthracis*, such as motility and resistance to the diagnostic y-phage. The subsequent genetic analysis placed the bacterium outside the *B. anthracis* lineage (Fig C) and evidenced the discovery of a novel anthrax-causing pathogen—*Bacillus cereus* biovar *anthracis* (*Bcbva*)—more than 100 years after the initial description of the first agent of anthrax (Klee et al, 2010). Since, *Bcbva* has been attributed to wildlife deaths across African rainforests, delineating it as a different ecotype to classical *B. anthracis*, which is typically savannah-associated, and it was therefore termed “rainforest anthrax”. Although *Bcbva*, like *B. anthracis*, has a broad host spectrum, it has been predominantly detected in primates and duikers, as well as western lowland gorillas. In TNP, *Bcbva* accounts for close to 40% of mammal mortality and threatens the overall survival of the local chimpanzee population (Hoffmann et al, 2017). The lethality in our closest relatives and serological human evidence indicate that *Bcbva* is zoonotic. Exposure of the local human population is probably facilitated via bushmeat, which highlights the importance of both public sensitization concerning risks associated with bushmeat handling and consumption, as well as strengthening diagnostic capabilities in *Bcbva* distribution areas to enable early treatment in case of spillover.

**Table:** 

*Box 1 Pathogen fact sheet:*	
Pathogen	*Bacillus cereus* biovar *anthracis (Bcbva)*
Host	Likely pan-mammalian (most cases detected in primates, including chimpanzees and gorillas, but also other rainforest species like duikers and forest elephants)
Reservoir	Environmental spore reservoirs established at carcass sites
Clinical signs	Previously healthy chimpanzees die acutely following a brief period of severe illness
Pathology	Hemorrhagic organs (foremost lungs); edemas
Transmission	Usually, non-contagious, spore uptake at spore-contaminated carcass sites, route of infection for wildlife remains elusive and might be species-dependent; flies might act as vectors
Detection	Histology, culture, PCR for *Bcbva* virulence markers
Significance	Relevant for public health (*Bcbva* is likely zoonotic) and species conservation (threatens chimpanzee communities)

**Figure:**
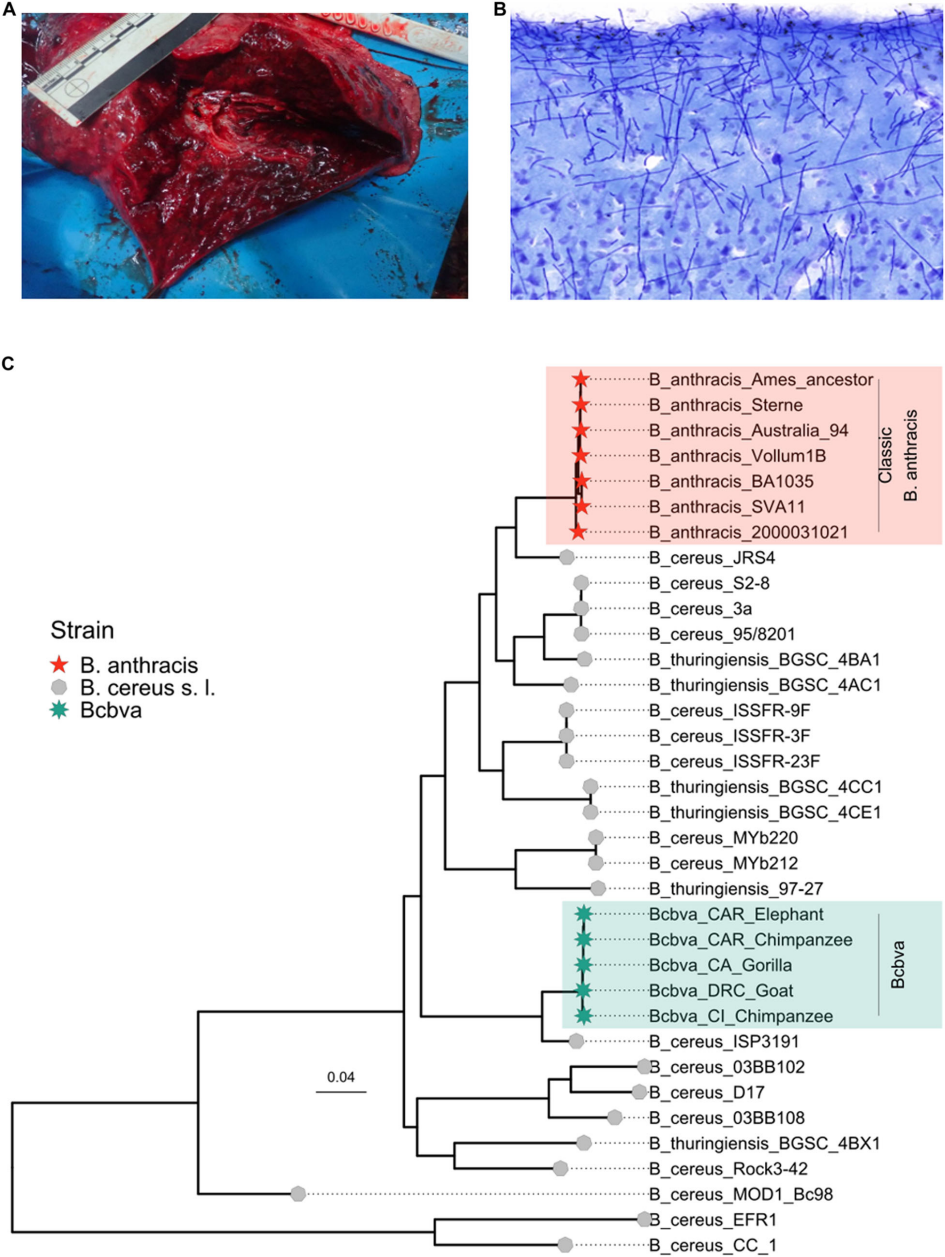
Box 1 (**A**) Severe hemorrhagic pneumonia in a wild chimpanzee caused by a *Bcbva* infection. (**B**) *Bcbva* invading and multiplying in a chimpanzee brain c) *Bcbva* ’s distinct phylogenetic placement outside the *B. anthracis* monophyly. Photo credit: Jenny Jaffe (**A**) and Carsten Jäger (**B**). Reproduced with permission.

Box 2 **Chronic skin disease** Reports of skin lesions and face deformities in wild great apes are not uncommon, and often infections with *Treponema pallidum* subsp. *pertenue* (TPE), the causative agent of yaws disease in humans, are suspected. Yaws-like disease in apes presents as ulcerative lesions that can lead to necrosis of facial soft tissue, cartilage, and bone. Lesions compatible with TPE infection have been observed in various sites across Sub-Saharan Africa, and molecular evidence from gorilla feces and chimpanzee bones suggested the presence of the pathogen in apes. Yet, the direct link between a chimpanzee with severe skin lesions and the molecular diagnosis of TPE was only possible in 2019, when a necropsy was performed on a wild chimpanzee that had to be euthanized in Guinea (Mubemba et al, 2020).

In 2018, colleagues studying wild, unhabituated chimpanzees in Canthanez National Park (CNP), Guinea Bissau, shared camera trap images of chimpanzees with severe skin lesions and facial deformations that looked distinct from yaws-like diseases. The animals presented with nodules, plaques, depigmentation, hair loss, and deformation of the hands reminiscent of advanced leprosy in humans. From the images collected over several years, it was possible to identify four affected individuals whose condition worsened over time. Around the same time, a habituated chimpanzee in Taï started showing similar leprosy-like lesions. The noninvasive nature of our work rendered direct sampling of the lesions impossible, but we detected *M. leprae* DNA in fecal samples and were able to generate a whole *M. leprae* genome.

At TNP, based on two decades of routine sampling of all habituated chimpanzees, we were able to target fecal samples of affected individuals through time. With the onset of clinical signs, fecal samples were consistently positive for *M. leprae* DNA. This finding prompted us to screen necropsy samples of 38 chimpanzees collected since 2000 for *M. leprae*. We identified a *M. leprae-*positive female chimpanzee who died in 2009 of a leopard attack. Retrospective analyses of photographs showed progressive depigmentation and other leprosy-like lesions, and molecular, serological and histopathological analyses confirmed *M. leprae* infection, unambiguously showing that chimpanzees can contract leprosy in the wild (Hockings et al, 2021). Both yaws and leprosy were long considered exclusively human diseases, and it remains to be investigated if apes are infected through contact to humans or potentially from an unknown animal or environmental reservoir. These findings show the value of noninvasive techniques, coupled with long-term health monitoring of habituated apes, and trained field veterinarians, to contribute to our understanding of the ecology of human pathogens.

**Table:** 

*Box 2 Pathogen fact sheet:*		
Pathogens	*Treponema pallidum* subsp. *pertenue*	*Mycobacterium leprae*
Host	Humans, chimpanzees, gorillas, monkeys	Humans, nine-banded armadillos, squirrels, chimpanzees
Reservoir	Humans, unknown animal reservoir?	Humans, unknown animal reservoir or environmental reservoir?
Symptoms	Ulcerative skin lesions, necrosis of soft tissue, cartilage, bone	Skin lesions including depigmentation, plaques, and nodules; hair loss, deformation of hands and feet, loss of nails
Transmission	Direct contact	Direct prolonged contact
Detection	Affected tissue, bone, and feces	In feces, necropsy samples, blood
Significance	Unknown	Unknown

**Figure:**
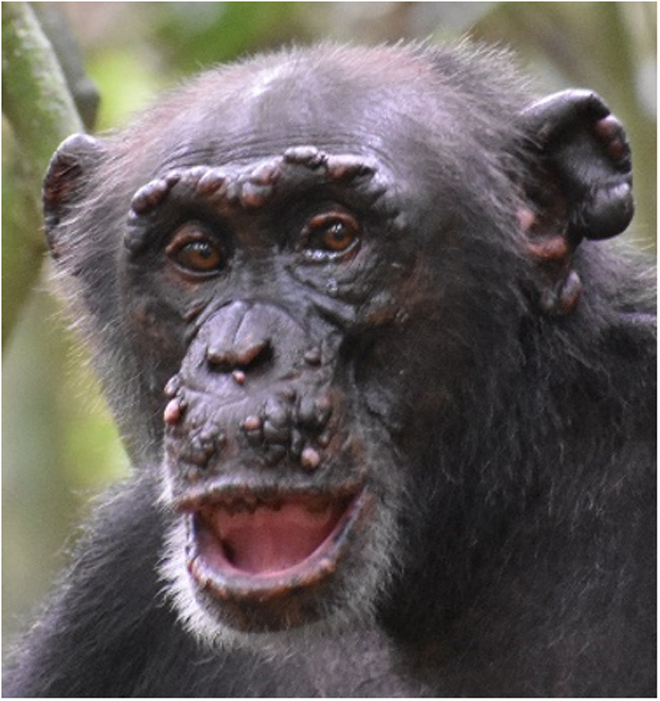
Box 2 Leprosy, with typical skin lesions, in a wild chimpanzee. Photo credit: Taï Chimpanzee Project. Reproduced with permission.

Box 3 **Mpox** Between 2017 and 2018, our team observed three temporally and spatially separate outbreak events in neighboring chimpanzee communities in Taï. During the first outbreak, the predominance of respiratory symptoms and the absence of diffuse skin lesions pointed towards a respiratory virus infection. However, during the subsequent two outbreaks, the presence of distinct pox lesions on affected individuals (Fig A) allowed for rapid identification of Mpox, formerly monkeypox, a viral infection similar to, but milder than the well-known, eradicated smallpox. The disease primarily presented with respiratory signs (labored breathing, nasal and ocular discharge, lethargy; Fig B) and some younger individuals developed severe illness, leading to one fatality. Our analysis of the non-invasively collected samples—feces, urine, saliva from chewed-up pieces of fruit and flies from around the chimpanzee group—showed not only the presence of Mpox virus (MPXV) DNA, but we were able to isolate viable MPXV from one fecal sample and one fly regurgitate.

These results suggest that the virus could be transmitted both by direct skin contact with infected individuals, and potentially through infection via feces and flies. Genomic analyses showed that the three distinct outbreaks correspond to separate transmission events originating from an unidentified reservoir host. Behavioral observation data collected during the outbreak showed a relationship between grooming behavior and the presence of MPXV in chimpanzee feces: individuals who exhibited a significantly lower grooming rate during the outbreak had no traces of MPXV in their feces. We also found that the overall grooming rates of affected groups decreased during the outbreak period, potentially having an impact on the evolution of the outbreak, which highlights the importance of recording behavioral observations during a disease outbreak. As longitudinal data in the TNP suggests, MXPV is endemic to the forest, but was absent in the past 30 years, re-emerging possibly due to a recent ecological shift within the habitat. Understanding the local ecological changes leading to these outbreaks and identifying the reservoir species could help us solve the puzzle of the recent emergence of Mpox in human populations.

**Table:** 

*Box 3 Pathogen fact sheet:*	
Pathogen	Mpox virus
Host	Humans, nonhuman primates, rodents
Reservoir	Unknown as of yet, rodents suspected
Symptoms	Maculo-papular (pox-like) lesions, respiratory signs, severe illness, death
Transmission	Direct and indirect transmission via respiratory droplets, body fluids, lesions, grooming; potentially indirect transmission via feces, flies
Detection	In noninvasive samples, such as fecal samples, urine, saliva, flies, and fly regurgitates with PCR
Significance	Respiratory-only manifestation in chimpanzees raises concern about the possibility of similar respiratory-only cases in humans that may be overlooked or misdiagnosed and which could contribute to the unnoticed transmission of the disease, leading to large-scale human outbreaks

**Figure:**
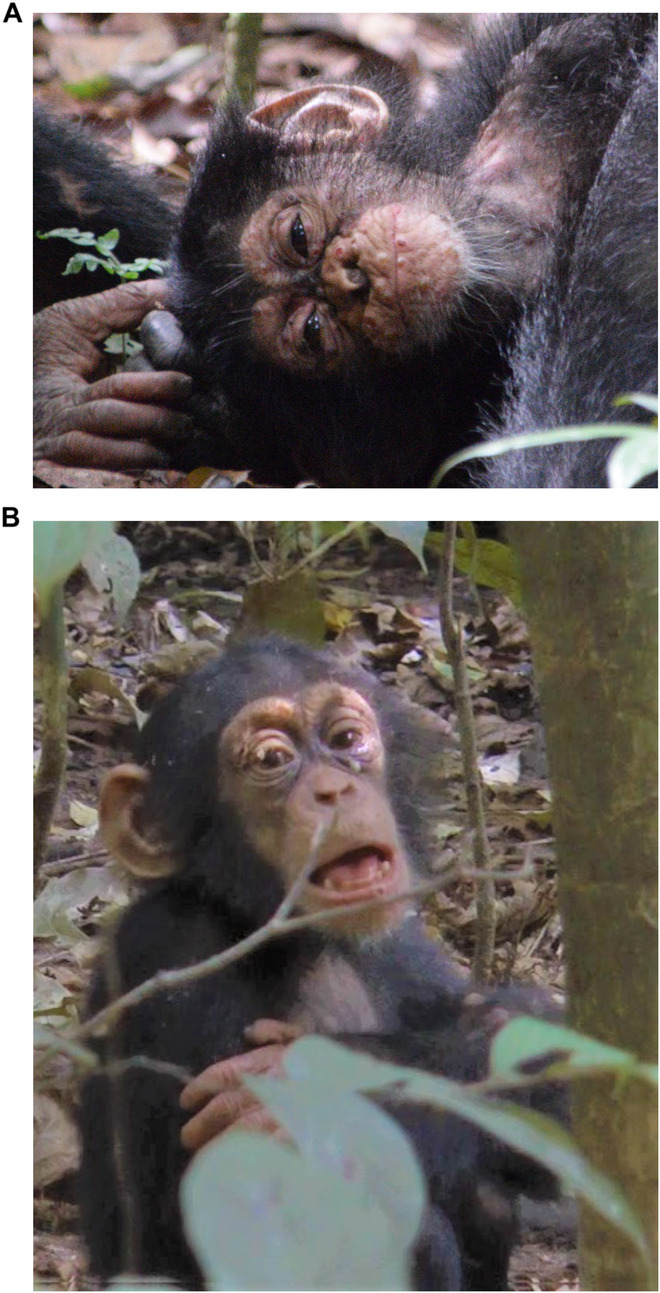
Box 3 (**A**) Classical Mpox manifestation with lesions on the 6th day after first observed signs of disease in an infant chimpanzee. (**B**) Predominantly respiratory manifestation: labored breathing in an infant chimpanzee. Photo credit: Taï Chimpanzee Project. Reproduced with permission.

Box 4 **Respiratory diseases** In May 1999, the entire north chimpanzee community (32 individuals at the time) in Taï manifested clinical signs such as elevated breathing rate, conspicuous breathing sounds, sneezing and coughing. The subsequent death of six individuals provided the first evidence of respiratory infections in wild, human-habituated great-ape populations (Köndgen et al, 2008). In the following years up to 2009, six additional outbreaks of respiratory disease occurred in the other two habituated communities, south and east. Morbidity was generally high (62–100%), and deaths across age groups occurred in four out of the six outbreaks.

Carcass sampling and PCR testing offered the first insights into the causative agents: common human respiratory viruses, such as the human orthopneumovirus and the human metapneumovirus. Viral infections were complicated by secondary bacterial colonization, which ultimately led to mortality. With the exception of one case in which *Pasteurella multocida* was co-detected, distinct strains of *Streptococcus pneumoniae* were found in all lethal outbreaks. Typing of pneumococcal strains was also suggestive of a human introduction.

The subsequent development of noninvasive diagnostics for respiratory pathogens in fecal samples offered the opportunity to investigate the two non-lethal outbreaks, and revealed once again the involvement of human pneumoviruses. Since these early reports, the majority of African great-ape research and tourism sites reported mild to lethal outbreaks caused by common human respiratory viruses, such as a human rhinovirus C, a human respirovirus type 3 and a human coronavirus OC43. This evidence led to the establishment of increasingly strict hygiene rules, ranging from adopting employee health programs, establishing a hygiene barrier in the camps to disinfect boots and change into clothes worn only in the forest, to keeping a distance and wearing a surgical mask when observing the great apes, up to establishing field laboratories for on-site testing prior to visiting great apes (Gilardi et al, 2015). The implementation of such measures significantly reduced the risk of pathogen introduction while maximizing the benefits of great-ape research and tourism projects, namely species and habitat protection.

A recent re-investigation of some of these outbreaks using genomic approaches highlighted how spillover of human viruses to great apes does not require mutations, nor does efficient spread within the groups, and that immunity against these pathogens is transient. Prevention through strict hygiene rules and human vaccination remains therefore the most powerful tool to reduce the risk of pathogen transmission. For details about these outbreaks and additional references, see Further Reading.

**Table:** 

*Box 4 Pathogen fact sheet:*	
Pathogens	Common human respiratory viruses and bacteria
Host	Chimpanzee, gorilla, bonobo, human
Reservoir	Humans
Symptoms	General malaise and respiratory signs; degree of severity depends on multiple factors related to the host, the virus, and the presence of secondary bacterial infections
Transmission	Respiratory droplets
Detection	In necropsy samples (lungs) and noninvasive samples (feces)
Significance	Leading cause of death

**Figure:**
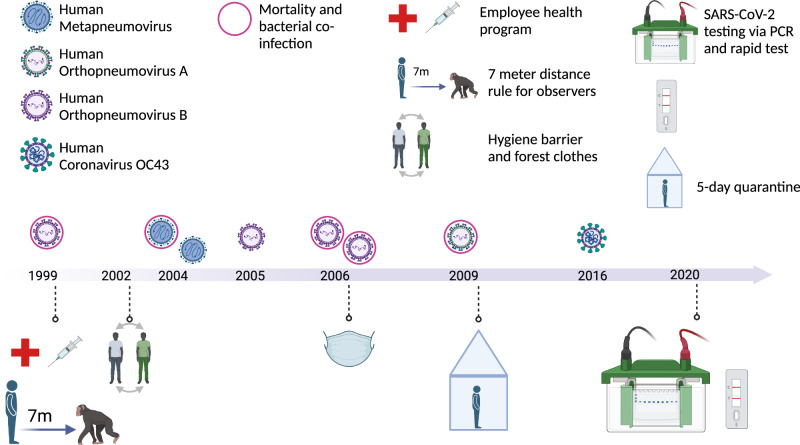
Box 4 Respiratory disease outbreaks in Taï chimpanzees and implementation of hygiene measures for staff and visitors (created with BioRender.com).

## Noninvasive approaches for monitoring great-ape health

**Figure 2:**
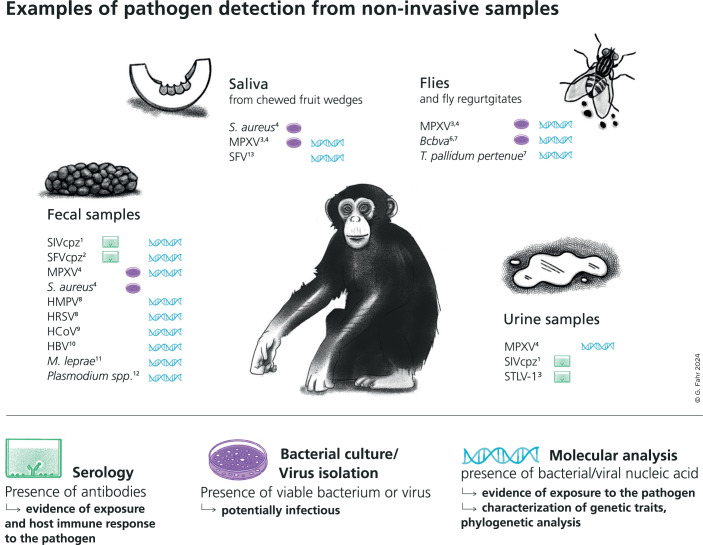
Noninvasive samples enable the detection of a variety of infectious agents. All examples of pathogens shown here were previously detected using noninvasive samples from primates, such as feces, urine, saliva or flies. This evidence has been obtained through serology, bacterial culture/virus isolation and/or molecular analysis, highlighting the potential and versatility of noninvasive samples for both health and biodiversity monitoring. Note that not detecting a pathogen does not necessarily imply its absence, and a large sample size is required to decrease the likelihood of a false negative result. For fecal samples, we included examples of pathogens not initially expected to be present in this sample type: fecally transmitted pathogens shed in feces in high copy numbers are not listed here. Examples presented here are based on unpublished results and published literature (see Further Reading).

As not all outbreaks and individual diseases observed in chimpanzees resulted in death, there was a clear need to develop noninvasive sampling approaches for detecting pathogens from materials that could be collected without disturbing the animals (Fig. 2). The discovery of the human immune-deficiency virus type 1 (HIV-1) in 1983 sparked a major search for the origins of this pathogen, which decisively advanced the development of such noninvasive techniques. Nonhuman primates were a prime suspect, and while the screening of captive great apes found HIV-1-positive chimpanzees, it was initially unclear if they had become infected by humans or if chimpanzees represented natural hosts for this virus. Pathogens transmitted through feces, such as gastrointestinal parasites, had long been studied using noninvasive samples, but Beatrice Hahn and colleagues developed methods to detect a systemic pathogen like HIV in noninvasive samples, in particular in feces from wild great apes. Armed with this tool, she contacted primatologists studying wild, human-habituated great apes and provided sampling kits with simple and clear instructions. This sampling ultimately led to a solid understanding of the zoonotic origins of HIV-1, while providing a clear indication that noninvasive detection of pathogens was possible, even beyond those that are fecally transmitted (Keele et al, 2006).

The TCP started regularly collecting fecal samples from chimpanzees, which have enabled the detection of the genetic material of a huge diversity of infectious agents, including adenoviruses, coronavirus, herpesviruses, paramyxoviruses, pneumoviruses, poxviruses, polyomaviruses, retroviruses (Calvignac-Spencer et al, 2021), *Mycobacterium leprae* (Hockings et al, 2021), *Streptococcus pneumoniae* and malaria parasites (Fig. 2). The presence of DNA or RNA from infectious agents with very different tropisms in feces can probably be explained by a combination of physiology and physiopathology—respiratory secretions are swallowed by chimpanzees—and behavior: skin lesions and wounds are investigated and often groomed by affected chimpanzees and their group mates. Initially, these explorations were largely PCR-based approaches targeting short genomic regions of the pathogen, but molecular advances and enrichment methods such as in solution hybridization capture now mean that full genomes can often be obtained from such materials, which allows for fine-grain analyses of many outbreaks (Boxes 1–4).

While nucleic acid detection and genome sequencing are a favorite tool to show the association between a disease and the presence of its causative agent in noninvasive samples, it is also increasingly possible to assess disease exposure using serology tools, as antibodies can sometimes be detected from feces (Fig. 2). Wildlife serology remains challenging for a number of reasons though, including a lack of species-specific conjugates, and the difficulties of finding appropriate positive and negative controls, as well as immunological cross reactivity. These challenges are exacerbated with noninvasive serological samples. However, technological advances that allow serological tools to be efficiently multiplexed and contrasted are improving their reliability (Ayouba et al, 2019).

While fecal samples are relatively easy to collect, technological innovations have opened up a diversity of other substrates for disease investigations. For example, biological anthropologists have long been interested in bones, which have recently revealed insights into the history of pathogens like *Treponema pallidum* subsp*. pertenue* and *Bcbva* in chimpanzees and other primates in Taï and elsewhere. To that end, it is ideal to have a trained veterinarian on site, even if it is a large resource investment. Accordingly, alternative sampling strategies for getting information about what killed an animal are making headway. For example, flies are found in large numbers on every carcass in the wild but also on skin lesions of living animals, taking up small amounts of biological material. By catching flies, either systematically to investigate the distribution of pathogens in an ecosystem, or in a targeted approach near symptomatic living animals or a carcass, we have been able to gain important insights into pathogen distribution and diversity.

## Understanding great-ape health in an ecological context

The observation of wild animals in their natural habitats also allows for a much richer understanding of how their ecology might influence the circulation of infectious agents, including toward humans. For example, most chimpanzees and bonobos hunt, creating frequent opportunities for zoonotic transmission. In Taï, this translates into chimpanzees and their favorite prey, red colobus monkeys, being infected with the Simian T-Lymphotropic Virus 1 (STLV-1) which is not species-specific, suggestive of frequent transmission (Leendertz et al, 2004b). On the other hand, while Simian immunodeficiency viruses (SIV) with strong host specificity infect sooty mangabeys (SIVsm), red colobus (SIVwrc) and olive colobus monkeys (SIVolc), chimpanzees do not appear to become infected with any of these viruses. The lack of SIV infections in these chimpanzees likely reflects a combination of intrinsic resistance and specific ecological interactions. Humans surrounding TNP hunt sooty mangabeys much more heavily than chimpanzees do and were the recipients of at least one transmission event, leading to a new lineage of the human immune-deficiency virus type 2 (HIV-2).

The observation of wild animals in their natural habitats also allows for a much richer understanding of how their ecology might influence the circulation of infectious agents…

Clearly understanding the range of species that great apes come into contact with represents an important frontier, particularly during outbreaks of novel pathogens for which the disease reservoir is poorly described, such as leprosy and Mpox (Boxes 2 and 3). Here again, molecular approaches can provide important additional insights into the diet: metabarcoding-based strategies coupled with chimpanzee blocking primers, revealed the dietary diversity of these animals. Continuous sampling of feces allows to explore the diet—and a potential source of infection—of a particular animal in the days and months prior to the eventual infection. In addition, environmental DNA-based approaches that detect the DNA animals leave behind offer new possibilities to describe animal biodiversity, providing opportunities to understand the communities in which these chimpanzees live.

These are instances where the field of disease ecology has benefited from collaborations with primatologists. However, these interdisciplinary approaches may in fact provide insights into great-ape behavior and evolution as well, which go beyond informing protection and conservation strategies. Indeed, infectious diseases represent a strong force of selection that has likely shaped primate behavior and life history traits. As the COVID-19 pandemic highlighted, behavior can have a critical impact on various aspects of a pathogen’s transmission. Great apes have species-specific social systems and cultures that are quite different from one another. For example, some live in large multi-male–multi-female groups (chimpanzee, bonobos, mountain gorilla), others in one-male-several-female groups (lowland gorillas), while others live more solitary lifestyles (orangutans). Bonobos and chimpanzees undergo fission–fusion dynamics, with individuals and subgroups breaking off for longer periods before coming together as a larger group again. Upon the death of the leading male, lowland gorilla communities disintegrate and surviving individuals join other groups, thereby creating opportunities for disease transmission. These diverse behaviors are predicted to play a major role in the dynamics of infectious diseases in different social systems and some of these differences may explain why certain highly virulent pathogens like Ebola viruses have only been observed in African great apes, and lowland gorilla populations with particularly devastating impacts, but not in orangutans.

It is also hypothesized that animals may modify their behavior during disease outbreaks. For example, during an Mpox outbreak in chimpanzees, animals groomed each other less, which may have reduced the spread of the pathogen in the social group (Patrono et al, 2020). Understanding the variation observed in the behavior of great apes, between species, between populations of the same species, within groups through time, or between individuals, provides exciting opportunities to explore links between behavior and disease, while potentially guiding intervention strategies.

## The African scientists’ perspective

Longstanding collaborations are a cornerstone of the success of these research efforts. African scientists and organizations represent an invaluable source of expertise and knowledge, but even when such expertise may not initially exist or be easily identified, there is a clear ethical imperative to contribute to developing or finding it. Many long-term research projects are conducted in regions with colonialist histories and great inequalities in terms of access to resources and training, which have created a clear need for benefit-sharing. Many countries also signed the Nagoya protocol, which defines the terms for collaboration and capacity building of local experts, and training of students is an essential pillar of these endeavors. This often involves training African staff and students in great-ape monitoring and sample collection techniques, as well as state-of-the-art molecular diagnostic methods, and giving them access to robust technical and analytical platforms for their research, for instance, in Western partner laboratories.

Many long-term research projects are conducted in regions with colonialist histories and great inequalities in terms of access to resources and training, which have created a clear need for benefit-sharing.

A major challenge is replicating this infrastructure in their home countries. Practically, we face challenges such as a lack of stable electricity, along with heat and humidity that are hard on infrastructure and machines. Because of the small market for laboratory machines and reagents, there is no local production and technical maintenance is extremely challenging. Coupled with higher shipping prices, this paradoxically makes equipping and maintaining laboratories more expensive despite much smaller national research budgets. However, these investments are worth it, as creating state-of-the-art labs in Africa generates jobs in these countries and allows for a network of South-South collaborations. On the other hand, African countries, despite their limited resources, should invest in research—including human resources and facilities —especially with regard to topics that can aid in the prevention and mitigation of emerging infectious diseases. Such prevention of infectious diseases would improve the well-being of the rural populations and ultimately reduce the disease burden and associated costs for these countries over the long-term.

African countries, despite their limited resources, should invest in research […] especially with regard to topics that can aid in the prevention and mitigation of emerging infectious diseases.

At the moment, many students and African experts must travel extensively as part of their careers and they face unique challenges that governments, funders and research institutions need to consider and address. We exemplarily highlight such travel-related challenges in African-German collaborations: Students must apply for a long-stay visa for visiting Germany, requiring embassy appointments that need to be booked months in advance. For citizens from Gabon and Central African Republic, for example, where Germany has no official diplomatic representation, this necessitates a preliminary stay in Cameroon to visit the sub-regional representation, which requires considerable upfront investments of time and funds. These processes are even more challenging for those with families they are trying to bring along. Visitors to Germany often need to make prohibitively large investments in advance, even if they may ultimately be reimbursed. There are also the challenges of language immersion, finding housing without a credit or local renting history, and increasing anti-immigrant sentiments in much of the Global North in addition to navigating access to childcare and the challenges of adapting wardrobes to different climates. Not least, during their fieldwork, but then again during their stays in Germany, doctoral students spend long periods without seeing their families.

## Conclusion

A sentinel approach, here explored through the lens of focusing on studies of just one family of primates—the great apes—has great potential to contribute to pandemic prevention and preparedness efforts. It enables us to identify pathogens that infect some of our closest relatives, as well as those that are frequently transmitted between species and that might thus represent potential zoonotic pathogens. In this way, a sentinel approach facilitates an understanding of which members of the vast microbial diversity should be prioritized for future studies and research endeavors. It can guide surveillance and efforts to mitigate spillover into human populations coming into contact with wildlife. There is still much to be discovered, as highlighted by the examples here, which resulted from more than two decades of disease surveillance at Taï. We are expanding our investigations into the sources of these pathogens that newly emerge in chimpanzees, aiming to gain a better understanding of their ecology and evolution.

The unique challenges faced by African scientists, whose cooperation is crucial to the success of such research efforts, must be considered in the planning, funding and execution of these investigations.

Numerous other long-term projects are also underway to study a diversity of wild animals, including other primates, carnivores, ungulates, bats, and rodents. These monitoring efforts will also provide important insights into microbial and pathogen communities, their transmission within species, sources of infection and the potential for spillover to other species, including humans. In all cases, these efforts require interdisciplinary collaboration and a solid foundation based on trust, acceptance of and respect for other disciplines, with clear benefit-sharing at both the local and global levels. The unique challenges faced by African scientists, whose cooperation is crucial to the success of such research efforts, must be considered in the planning, funding and execution of these investigations. Although overcoming these obstacles and familiarizing oneself with the language and concerns of other disciplines can be challenging, leveraging synergies can maximize the use of these unique opportunities, building upon the huge investments of time and energy dedicated to these projects. We hope that such collaborations will become a best practice for scientists studying wildlife (Box 5).

Box 5 **Further Reading** *Field necropsy protocol:*

Gräßle T, Crockford C, Eichner C, Girard-Buttoz C, Jäger C, Kirilina E, Lipp I, Düx A, Edwards L, Jauch A et al (2023) Sourcing high tissue quality brains from deceased wild primates with known socio-ecology. Methods Ecol Evol 14:1906–1924

*Postmortem pathogen detection (Fig. 1):*

^1^ Prescott J, Bushmaker T, Fischer R, Miazgowicz K, Judson S, Munster VJ (2015) Postmortem stability of Ebola virus. Emerg Infect Dis 21:856

^2^ Gogarten JF, Düx A, Schuenemann VJ, Nowak K, Boesch C, Wittig RM, Krause J, Calvignac-Spencer S, Leendertz FH (2016) Tools for opening new chapters in the book of *Treponema pallidum* evolutionary history. Clin Microbiol Infect 22:916–921

^3^ Hoffmann et al (2017) (see main text references)

^4^ Calvignac S, Terme J-M, Hensley SM, Jalinot P, Greenwood AD, Hänni C (2008) Ancient DNA identification of early 20th century simian T-cell leukemia virus type 1. Mol Biol Evol 25:1093–1098

^5^ Suzuki K, Takigawa W, Tanigawa K, Nakamura K, Ishido Y, Kawashima A, Wu H, Akama T, Sue M, Yoshihara A et al (2010) Detection of *Mycobacterium leprae* DNA from archaeological skeletal remains in Japan using whole genome amplification and polymerase chain reaction. PLoS ONE 5:e12422

^6^ Lee OYC, Wu HHT, Donoghue HD, Spigelman M, Greenblatt CL, Bull ID, Rothschild BM, Martin LD, Minnikin DE, Besra GS (2012) *Mycobacterium tuberculosis* complex lipid virulence factors preserved in the 17,000-year-old skeleton of an extinct bison, *Bison antiquus*. PLoS ONE 7:e41923

*Non-invasive sampling (Fig. 2):*

^1^ Sharp PM, Shaw GM, Hahn BH (2005) Simian immunodeficiency virus infection of Chimpanzees. J Virol 79:3891–3902

^2^ Liu W, Worobey M, Li Y, Keele BF, Bibollet-Ruche F, Guo Y, Goepfert PA, Santiago ML, Ndjango J-BN, Neel C et al (2008) Molecular ecology and natural history of simian foamy virus infection in wild-living chimpanzees. PLoS Pathog 4:e1000097

^3^ Leendertz FH, Boesch C, Ellerbrok H, Rietschel W, Couacy-Hymann E, Pauli G (2004) Non-invasive testing reveals a high prevalence of simian T-lymphotropic virus type 1 antibodies in wild adult chimpanzees of the Tai National Park, Cote d’Ivoire. J Gen Virol 85:3305–3312

^4^ Patrono et al (2020) (see main text references)

^5^ Schaumburg F, Mugisha L, Kappeller P, Fichtel C, Köck R, Köndgen S, Becker K, Boesch C, Peters G, Leendertz FH (2013) Evaluation of noninvasive biological samples to monitor *Staphylococcus aureus* colonization in great apes and lemurs. PLoS ONE 8:e78046

^6^ Hoffmann et al (2017) (see main text references)

^7^ Gogarten JF, Düx A, Mubemba B, Pléh K, Hoffmann C, Mielke A, Müller‐Tiburtius J, Sachse A, Wittig RM, Calvignac‐Spencer S et al (2019) Tropical rainforest flies carrying pathogens form stable associations with social nonhuman primates. Mol Ecol 28:4242–4258

^8^ Patrono et al (2022) (see Further Reading Respiratory diseases)

^9^ Patrono et al (2018) (see Further Reading Respiratory diseases)

^10^ Makuwa M, Souquiere S, Clifford SL, Mouinga-Ondeme A, Bawe-Johnson M, Wickings EJ, Latour S, Simon F, Roques P (2005) Identification of hepatitis B virus genome in faecal sample from wild living chimpanzee (*Pan troglodytes troglodytes*) in Gabon. J Clin Virol 34:S83–S88

^11^ Hockings et al (2021) (see main text references)

^12^ Plenderleith LJ, Liu W, Li Y, Loy DE, Mollison E, Connell J, Ayouba A, Esteban A, Peeters M, Sanz CM et al (2022) Zoonotic origin of the human malaria parasite *Plasmodium malariae* from African apes. Nat Commun 13:1868

^13^ Smiley Evans T, Barry PA, Gilardi KV, Goldstein T, Deere JD, Fike J, Yee J, Ssebide BJ, Karmacharya D, Cranfield MR et al (2015) Optimization of a novel noninvasive oral sampling technique for zoonotic pathogen surveillance in nonhuman primates. PLoS Negl Trop Dis 9:e0003813

*Respiratory diseases (Box 4):*

Grützmacher K, Keil V, Leinert V, Leguillon F, Henlin A, Couacy-Hymann E, Köndgen S, Lang A, Deschner T, Wittig RM et al (2018) Human quarantine: Toward reducing infectious pressure on chimpanzees at the Taï Chimpanzee Project, Côte d’Ivoire. Am J Primatol 80:e22619

Köndgen S, Schenk S, Pauli G, Boesch C, Leendertz FH (2010) Noninvasive monitoring of respiratory viruses in wild chimpanzees. EcoHealth 7:332–341

Köndgen S, Leider M, Lankester F, Bethe A, Lübke-Becker A, Leendertz FH, Ewers C (2011) *Pasteurella multocida* involved in respiratory disease of wild chimpanzees. PLoS ONE 6:e24236

Köndgen S, Calvignac-Spencer S, Grützmacher K, Keil V, Mätz-Rensing K, Nowak K, Metzger S, Kiyang J, Lübke-Becker A, Deschner T et al (2017) Evidence for human *Streptococcus pneumoniae* in wild and captive chimpanzees: a potential threat to wild populations. Sci Rep 7:14581

Patrono LV, Röthemeier C, Kouadio L, Couacy‐Hymann E, Wittig RM, Calvignac‐Spencer S, Leendertz FH (2022) Non‐invasive genomics of respiratory pathogens infecting wild great apes using hybridisation capture. Influenza Other Respir Viruses 16:858–861

Patrono LV, Samuni L, Corman VM, Nourifar L, Röthemeier C, Wittig RM, Drosten C, Calvignac-Spencer S, Leendertz FH (2018) Human coronavirus OC43 outbreak in wild chimpanzees, Côte d’Ivoire, 2016. Emerg Microbes Infect 7:1–4

### Supplementary information

Peer Review File

## Glossary

*Bcbva*: *Bacillus cereus* biovar *anthracis*, causing agent of rainforest anthrax
CNP: Canthanez National Park, Guinea Bissau
EBOV: Ebola virus
EIDs: Emerging infectious diseases
Grooming: Normal primate behavior involving physical cleaning, inspection, and manipulation of each other’s fur and skin
Habituated animals: Wildlife in their natural habitat, but accustomed to the presence of humans
HBV: Hepatitis B Virus
HCoV: Human Corona Virus
HIV-1, HIV-2: Human Immune-deficiency Virus type 1, type 2
HMPV: Human Metapneumovirus
HRSV: Human Respiratory Syncytial Virus
IUCN: International Union for Conservation of Nature
Mpox (MPXV): Formerly called ‘monkeypox’, infectious disease caused by the monkeypox virus, MPXV
*Mycobacterium leprae*: bacterial pathogen and causative agent of leprosy, chronic infectious disease
NHPs: Non-human primates, that is: monkeys and apes
Non-invasive sampling: sampling without disturbing the animals
One Health concept: The idea that human, animal, and environmental health are inextricably intertwined
SFV: Simian Foamy Virus
SIV: Simian immunodeficiency viruses; a species-specific pathogen, e.g., SIVcpz (infects chimpanzee)
STLV-1: Simian T-Lymphotropic Virus 1
TAFV: Taï Forest ebolavirus
TCP: Taï Chimpanzee Project
TNP: Taï National Park, Côte d’Ivoire
*Treponema pallidum* (TPE): bacterial pathogen and causative agent of yaws, a chronic skin infection
