# Supplementary material for: An ounce of prevention is better: Monitoring wildlife health as a tool for pandemic prevention
Source: EMBO Rep. 2024 Jun 7;25(7):2819–31. doi: 10.1038/s44319-024-00156-z (PMC11239677; doi:10.1038/s44319-024-00156-z)
Supplement: Supplementary file 1 — Peer Review File [file 44319_2024_156_MOESM1_ESM.pdf]

EMBO reports

An ounce of prevention is better

Fabian Leendertz, Jan Gogarten, Ariane Düx, Tobias Gräßle, Christelle Lumbu, Stephanie Markert, Livia Patrono, Kamilla Pléh, Frederic Niatou Singa, Coch Tanga, Thais Tombolomako, Emmanuel Couacy-Hymann, Leonce Kouadio, Steve Ahuka-Mundeke, Patrice Makouloutou-Nzassi, and Sébastien Calvignac-Spencer

Corresponding author(s): Fabian Leendertz (fabian.leendertz@helmholtz-hioh.de)

Review Timeline:

Submission Date:	7th Mar 24
Accepted:	30th Apr 24

Editor: Holger Breithaupt

Transaction Report: This article was editorially reviewed.
